# Temporalis muscle thickness as a biomarker for sarcopenia: correlation with appendicular skeletal muscle mass in healthy older adults

**DOI:** 10.1002/alz70856_101748

**Published:** 2025-12-24

**Authors:** Andrew Huynh, Scott Wrigley, James Andrews, Belinda M. Brown, Sanka Amadoru, Qiongshi Lu, Ting Ye, Jeremiah J Peiffer, Paul K Crane, Christopher C. Rowe, Louise Burrell, Paul A Yates

**Affiliations:** ^1^ Austin Health, Melbourne, VIC, Australia; ^2^ The Florey Institute of Neuroscience and Mental Health, Melbourne, VIC, Australia; ^3^ University of Melbourne, Melbourne, VIC, Australia; ^4^ University of Alabama, Birmingham, AL, USA; ^5^ Murdoch University, Perth, Western Australia, Australia; ^6^ Alzheimer's Research Australia, Perth, Western Australia, Australia; ^7^ Edith Cowan University, Perth, Western Australia, Australia; ^8^ University of Wisconsin‐Madison, Madison, WI, USA; ^9^ University of Washington, Seattle, WA, USA; ^10^ Kaiser Permanente Washington Health Research Institute, Seattle, WA, USA; ^11^ Austin Health, Heidelberg, VIC, Australia

## Abstract

**Background:**

Sarcopenia, an age‐related loss of skeletal muscle strength, mass and function, is linked with dementia and Alzheimer's disease (AD). Current guideline‐recommended tools to diagnose sarcopenia, such as appendicular skeletal muscle mass (ASM), calculated as the sum of lean muscle mass in the arms and legs via dual‐energy X‐ray absorptiometry (DXA), are not commonly done in AD studies. However, brain magnetic resonance imaging (MRI) is regularly performed in AD studies, and temporalis muscle thickness (TMT) has been suggested as a potential sarcopenia biomarker. As a first step in evaluating whether TMT could be a useful sarcopenia diagnostic tool, we aimed to ascertain if TMT correlates with ASM in healthy older adults.

**Method:**

We conducted a retrospective study of healthy cognitively‐unimpaired older adults in the Intense Physical Activity and Cognition study, in whom MRI and DXA had been performed on the same visit. TMT was measured on axial T1‐weighted MRIs bilaterally perpendicular to the long‐axis of the temporalis muscle using the orbital roof and Sylvian fissure as anatomical landmarks, and average TMT used for analysis. ASM was adjusted for body size (height^2^). Sarcopenia was defined as ASM< 7.0 kg/m^2^ for males and <5.5 kg/m^2^ for females as per the 2019 European working group on sarcopenia in older people criteria. Pearson correlation assessed the relationship between TMT and ASM or age.

**Result:**

There were 95 participants (mean±standard deviation [SD] age 69.1±5.2 years, 53% female, median Montreal Cognitive Assessment score 27 [Interquartile range 25 – 28],11% had sarcopenia). The mean±SD ASM was 7.0±1.2 kg/m^2^ and TMT was 7.3±1.2 mm. TMT and ASM were moderately correlated (*r* = 0.41, 95% confidence interval 0.23 – 0.56). TMT did not correlate with age but differed significantly between those with (mean±SD 7.4±1.2) and without sarcopenia (mean±SD 6.2±0.8, *p* = 0.004).

**Conclusion:**

Among a cohort of cognitively‐unimpaired older adults, TMT demonstrated moderate correlation with ASM. While futher studies are needed, these findings suggest that MRI‐based assessement of TMT could be a practical tool to diagnose sarcopenia in AD studies. Future studies in AD patients should explore the relationship between TMT and long‐term clinical and functional outcomes.

